# Combination of MALDI-TOF Mass Spectrometry and Machine Learning for Rapid Antimicrobial Resistance Screening: The Case of *Campylobacter* spp.

**DOI:** 10.3389/fmicb.2021.804484

**Published:** 2022-02-18

**Authors:** Maureen Feucherolles, Morgane Nennig, Sören L. Becker, Delphine Martiny, Serge Losch, Christian Penny, Henry-Michel Cauchie, Catherine Ragimbeau

**Affiliations:** ^1^Environmental Research and Innovation (ERIN) Department, Luxembourg Institute of Science and Technology, Belval, Luxembourg; ^2^Laboratoire National de Santé, Epidemiology and Microbial Genomics, Dudelange, Luxembourg; ^3^Institute of Medical Microbiology and Hygiene, Saarland University, Homburg, Germany; ^4^Swiss Tropical and Public Health Institute, Basel, Switzerland; ^5^University of Basel, Basel, Switzerland; ^6^National Reference Centre for Campylobacter, Laboratoire des Hôpitaux Universitaires de Bruxelles-Universitaire Laboratorium Brussel (LHUB-ULB), Brussels, Belgium; ^7^Université de Mons (UMONS), Mons, Belgium; ^8^Laboratoire de Médecine Vétérinaire de l’Etat, Dudelange, Luxembourg; ^9^Chambre des Députés du Grand-Duché de Luxembourg, Parliamentary Research Service, Luxembourg, Luxembourg

**Keywords:** MALDI-TOF MS, antimicrobial resistance screening, AMR, machine learning, *Campylobacter*, diagnostics

## Abstract

While MALDI-TOF mass spectrometry (MS) is widely considered as the reference method for the rapid and inexpensive identification of microorganisms in routine laboratories, less attention has been addressed to its ability for detection of antimicrobial resistance (AMR). Recently, some studies assessed its potential application together with machine learning for the detection of AMR in clinical pathogens. The scope of this study was to investigate MALDI-TOF MS protein mass spectra combined with a prediction approach as an AMR screening tool for relevant foodborne pathogens, such as *Campylobacter coli* and *Campylobacter jejuni*. A One-Health panel of 224 *C. jejuni* and 116 *C. coli* strains was phenotypically tested for seven antimicrobial resistances, i.e., ciprofloxacin, erythromycin, tetracycline, gentamycin, kanamycin, streptomycin, and ampicillin, independently, and were submitted, after an on- and off-plate protein extraction, to MALDI Biotyper analysis, which yielded one average spectra per isolate and type of extraction. Overall, high performance was observed for classifiers detecting susceptible as well as ciprofloxacin- and tetracycline-resistant isolates. A maximum sensitivity and a precision of 92.3 and 81.2%, respectively, were reached. No significant prediction performance differences were observed between on- and off-plate types of protein extractions. Finally, three putative AMR biomarkers for fluoroquinolones, tetracyclines, and aminoglycosides were identified during the current study. Combination of MALDI-TOF MS and machine learning could be an efficient and inexpensive tool to swiftly screen certain AMR in foodborne pathogens, which may enable a rapid initiation of a precise, targeted antibiotic treatment.

## Introduction

Antimicrobial susceptibility testing (AST) is a key technology in diagnostic microbiology and is essential for a targeted treatment and to limit the widespread use of broad-spectrum antibiotics. Over the past decades, many improvements have helped to accelerate, standardize, and harmonize testing facilities, e.g., through the implementation of automated and semi-automated devices combining identification and AST (e.g., Vitek 2^®^), using optical systems for measuring changes in bacterial growth and determining antimicrobial susceptibility, and using rapid diagnostic tests for same-day AST results (Mitchell and Alby, 2017; Benkova et al., 2020; Roth et al., 2021). In a concern for harmonization, disk-diffusion and microdilution antibiograms, recommended by the European committee on antimicrobial susceptibility testing (EUCAST, human medicine) or the European food safety authority (EFSA, veterinary medicine), are still the reference methods for determination of antimicrobial resistances (AMR). These tests are based on bacterial growth, requiring between 16 and 24 h for rapid growing pathogens and longer for fastidious pathogens (e.g., mycobacteria and *Helicobacter pylori*) (Barlam et al., 2016; Arena et al., 2017). Results are usually qualitative and classed into categories, i.e., susceptible or resistant, depending on the breakpoint calibrated by the EUCAST, or expressed as minimum inhibitory concentration (MIC) (Benkova et al., 2020). While these conventional methods are effective, they are cumbersome, time-consuming, and do not enable the rapid choice of an effective targeted anti-infective treatment. Yet, development of “fast microbiology” technologies or rapid diagnostic tests, including Matrix assisted laser desorption/ionization time of flight mass spectrometry (MALDI-TOF MS), results in the improvement of the antimicrobial stewardship by decreasing the “patient–physician” workflow before treatment (Bookstaver et al., 2017; Mangioni et al., 2019).

MALDI-TOF MS is a soft-ionized mass spectrometry method developed as an analytical tool to identify and understand the structure of unknown biomolecules (Gibson and Costello, 2000). In an evolving field, this automatic technique became the reference method for identifying microorganisms such as bacteria (Clark et al., 2013; Singhal et al., 2015), mycobacteria (Rodriguez-Granger et al., 2018; Rotcheewaphan et al., 2019) and fungi (Florio et al., 2018; Robert et al., 2021). The resolution power of the system operates at the species level and even at sub-species level for a number of pathogens in clinical microbiology (Fall et al., 2015; Feucherolles et al., 2021). It is a fast and cost-efficient process, with a positive impact on public health analytical pipelines (Ge et al., 2017; Rodríguez-Sánchez et al., 2019). Identification of other organisms, like protozoa (Del Chierico et al., 2016), helminths (Bredtmann et al., 2017; Feucherolles et al., 2019b; Sy et al., 2021; Wendel et al., 2021), viruses (Iles et al., 2020; Rybicka et al., 2021), and arthropods (Tahir et al., 2017; Boucheikhchoukh et al., 2018; Tandina et al., 2018), is also feasible in a research context. However, only the routine identification part of the diagnostics workflow is currently carried out by MALDI-TOF MS.

Over the last 5 years, machine learning (ML), a subset of artificial intelligence, has gained interest in many areas of research pertaining to an improved diagnosis of diseases (e.g., cancer detection, infectious diseases, etc.) (Caballé et al., 2020; Goodswen et al., 2021; Nami et al., 2021). This popularity is greatly explained by the current era, where large daily amounts of data are being collected digitally, known as big data, which are requiring new approaches to investigate it. Mass spectra are routinely generated by MALDI-TOF MS and most of the time not exploited for additional analysis beyond the sole identification of microorganisms. Even if several reports highlighted successful applications of MALDI-TOF MS for detection of bacterial AMR, by the presence of specific biomarkers (Feucherolles et al., 2019a; Oviaño and Bou, 2019; Yoon and Jeong, 2021) identified by classical statistical methods, there is still a mine of information encrypted in the mass spectra. More recently, a growing number of reports combining MALDI-TOF mass spectrometry and ML have shown promising results for clinical big data problems, such as AMR screening (Weis et al., 2020a,b). The majority of these studies used pathogens such as *Staphylococcus aureus* and the β-lactam antibiotic family (Sogawa et al., 2017; Wang et al., 2018; Tang et al., 2019). Therefore, there are very few published data concerning other relevant clinical or foodborne pathogens or antimicrobials such as the quinolones (e.g., ciprofloxacin) and macrolides (e.g., erythromycin and azithromycin) (Sabença et al., 2020; Sousa et al., 2020). However, macrolides and quinolones are frontline antibiotics used to treat severe infectious gastroenteritis and categorized by the World Health Organization (WHO) as critically important in human medicine (WHO, 2019).

Campylobacteriosis, mainly caused by *C. jejuni* and *C. coli*, is the main global cause of bacterial gastroenteritis in humans (Chlebicz and Śliżewska, 2018). Likewise, 10.9 and 0.6% of *C. coli* and *C. jejuni*, respectively, isolated from humans were multi-resistant to ciprofloxacin, erythromycin, tetracycline, and gentamycin in 2019 (EFSA and ECDC, 2021). In food-producing animals, 26.9% of *C. coli* isolated from calves were resistant to at least three of the previously cited antimicrobials. MALDI-TOF MS already has been applied for proteo-typing of *C. coli*, *C. fetus*, and more recently for *C. concisus* genomospecies (Emele et al., 2019a,b; On et al., 2021). Also, its ability to distinguish β-lactam-resistant strains from sensitive ones by pre-processing mass spectra before analysis was reported (Penny et al., 2016). However, there are no published reports concerning the direct application of the mass spectrometry and ML for direct prediction of AMR in *Campylobacter* spp.

Therefore, the aim of this study is to show that MALDI-TOF MS combined with an ML approach could be a useful tool for a fast and precise AMR screening of relevant foodborne pathogens, such as *C. coli* and *C. jejuni*. While campylobacteriosis is mainly self-limiting and do not require specific antibiotherapy, such a combination strategy may aid to swiftly prescribe a definitive antimicrobial therapy and therefore limit an empirical broad-spectrum strategy for other pathogens. ML prediction based on protein mass spectra will be investigated at the species-specific and antibiotic resistance level. The impact of different protein extraction methods, i.e., on- and off-plate extraction, on resistance predictions will also be considered.

## Materials and Methods

### Campylobacter Collection

#### Strains

A One-Health collection of 224 *C. jejuni* and 116 *C. coli* isolates, obtained from humans (*n* = 226), in environmental samples, i.e., surface water (*n* = 33), and animals including wild life: raccoons (*n* = 8), wild birds (*n* = 17), and cattle, i.e., bovine (*n* = 20), pig (*n* = 1), and poultry (*n* = 35), were used in the current study.

Antimicrobial resistances patterns were established by disk diffusion antibiograms for fluroquinolones [ciprofloxacin (Cip, 5 μg)], macrolides [erythromycin (Ery, 15 μg)], tetracyclines [tetracycline (Tet, 30 μg)], aminoglycosides [gentamycin (Gent, 10 μg), kanamycin (Kana, 30 μg), Streptomycin (Strep, 10 μg)], and β-lactams [ampicillin (Amp, 10 μg)] following the French Microbiology Society (SFM) and EUCAST recommendations (Recommendations 2020 v1.1 April) resulting in patterns addressed in Table 1. For antibiotics not described for *Campylobacter* spp., i.e., kanamycin and streptomycin, EUCAST recommendation for the *Enterobacterales* group was applied. The latter was added to the study based on ResFinder analysis by using Whole Genome Sequencing (WGS) data (Bortolaia et al., 2020). The Lys43Arg mutation in the *rspL* gene as well as *ant*(6) and *aadE* genes and conferring the streptomycin resistance were detected (Olkkola et al., 2010; Fabre et al., 2018). Likewise, the *aph*(3) gene conferring among other kanamycin resistance was detected (Fabre et al., 2018). The phenotypic details of the collection are described in Supplementary File 1.

**TABLE 1:** 

**(A)** Antimicrobial susceptibility patterns of *Campylobacter* isolates used in the present study.			
		**Resistant isolates**	
**Antibiotic classes**	**Antibiotics**	***C. jejuni* (*n* = 224)**	***C. coli* (*n* = 116)**

	Susceptible (S)*	70 (31.2%)	25 (21.6%)
Fluroquinolones	*Ciprofloxacin (Cip)*	123 (54.9%)	60 (51.7%)
Macrolides	*Erythromycin (Ery)*	2 (0.9%)	31 (26.7%)
Tetracyclines	*Tetracycline (Tet)*	90 (40.2%)	70 (60.3%)
Aminoglycosides	*Gentamycin (Gent)*	1 (0.4%)	11 (9.5%)
	*Kanamycin (Kana)*	18 (8.0%)	18 (15.5%)
	*Streptomycin (Strep)*	11 (4.9%)	35 (30.2%)
B eta-Lactams	*Ampicillin (Amp)*	90 (40.2%)	58 (50.0%)

**(B)** Diversity of antimicrobial resistance pattern in the collection.			

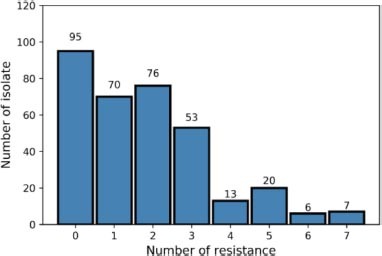			

*Susceptible to all tested antimicrobials.

#### Growth Conditions

All strains were inoculated on chocolate agar plates (Thermo Scientific, Waltham, MA, United States) with -80°C stock suspension stored in FBP medium complemented with *Campylobacter* growth supplement (Thermo Fisher Scientific), and incubated for 48 ± 2 h at 42°C under micro aerobic conditions using CampyGen 2.5 L gas packs (Thermo Fisher Scientific).

### Matrix Assisted Laser Desorption/Ionization Time of Flight Mass Spectrometry Analysis

#### Sample Preparation

For every biological assay, an off- and on-plate extraction and a direct deposit were performed. For the off-plate or also known as ethanol/formic acid protein extraction (EtOH/ACN), bacteria were suspended in 300 μl milliQ water and 900 μl absolute ethanol (Merck, Darmstadt, Germany). The mix was centrifuged for a further 2 min and the residual ethanol was discarded. A total of 25 μl for both 70% (v/v) formic acid (Merck, Darmstadt, Germany) and acetonitrile (Merck) were mixed up to the dry pellet. A final centrifugation was performed, and then 1 μl of supernatant was spotted onto a one-use MALDI Biotarget (96 targets; Bruker Daltonics GmbH, Bremen, Germany). For the formic acid on-plate extraction (FA), a smear of a bacteria colony is directly carried out on the biotarget and then overlayed with a 1 μl 70% formic acid. For the direct deposit, a bacteria colony is directly streaked on the biotarget. For all deposits and extractions, as soon as the sample was dried, the spot was overlaid with 1 μl of portioned HCCA matrix solution (Bruker Daltonics GmbH) prepared with standardized acetonitrile 50%, water 47.5%, and trifluoroacetic acid 2.5% solution (Sigma-Aldrich, Saint Louis, MO, United States). Bruker bacterial test standard (BTS) was used for an external calibration of the apparatus.

For each method of extraction, three independent cultures (biological replicates) on three different days (reproducibility) were performed. Each biological replicate was spotted thrice (technical replicates) on the same day (repeatability), resulting in nine spectra per isolate.

#### Data Acquisition

MALDI-TOF MS analysis was performed using a Biotyper Microflex LT/SH (Bruker Daltonics GmbH) by using the AutoXecute acquisition method (MBT_AutoX) in FlexControl software v3.4., with a 2–20 kDa mass-to-charge ratio (*m/z*) range in a positive linear mode. Before measurement, the system was calibrated using the automatic calibration feature with the BTS. For each sample spot, an automatic acquisition with 240 laser shots was performed.

#### Mass Spectra Analysis

All protein spectra were identified by using the BDAL Bruker database (*n* = 8,468 MSPs), containing at least 3,000 different bacterial and fungi species, through the MBT Compass Explorer interface (v.4.1). The software attributed a log score value between 0 and 3.00. A score between 0 and 1.69 was considered as a not reliable identification. A score between 1.70 and 1.99 was considered as probable genus identification and scores from 2.00 to 2.29 as reliable genus identification and a probable species identification. Finally, a score between 2.30 and 3.00 was deemed as highly probable species identification.

Then, spectra were uploaded on FlexAnalysis v3.0 (Bruker Daltonics GmbH) and an internal calibration was carried out on the 4,365 *m/z* peak, identified as a 50 S ribosomal protein L36 by Zautner et al. (2016) in *Campylobacter*, which is shared by all samples and the BTS. Mass spectra were converted into mzML files and imported into BioNumerics v7.6 software platform (BioMérieux, Craponne, France). Spectra were pre-processed using the workflow described by Penny and collaborators [binned baseline (size = 77), Kaiser Window (size = 33), Moving bar (width = 129)], with a sound-to-noise ratio threshold of 10 (Penny et al., 2016). The peak detection parameters were the following: Continuous wavelet transformation (CWT) ridges, double peaks, and a relative intensity of 2%. Biological replicate spectra were summarized to create an average spectrum, or Main Spectra Profile (MSP), per isolate and extraction. Finally, a peak matching was performed on MSPs, resulting in 91 peaks.

### Machine Learning Analysis

#### Pre-processing

**FIGURE 1:**
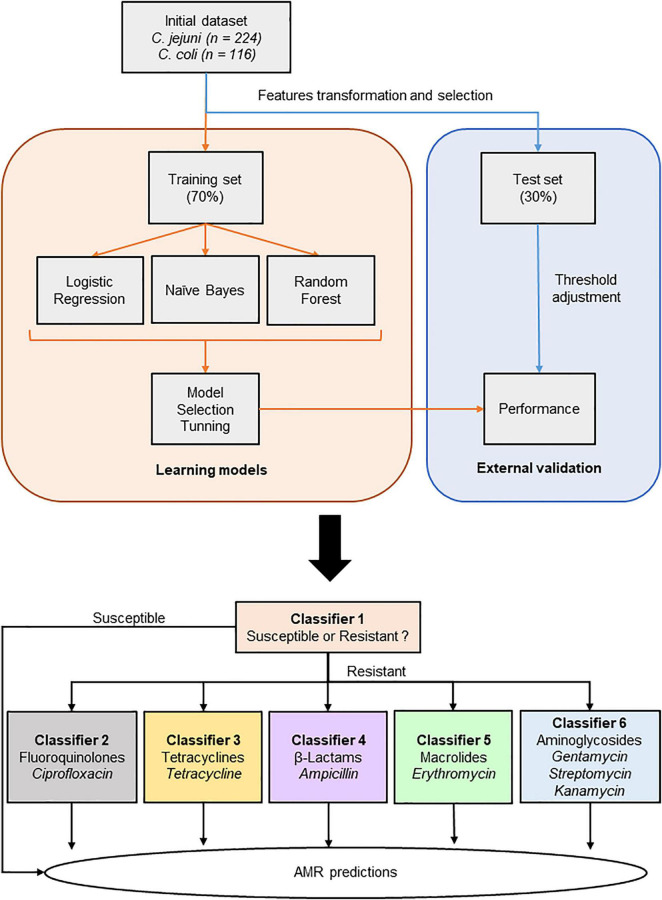
Schematic overview of the machine learning workflow.

Tables including intensity values of the peak matching MSPs for the three types of extraction were exported into csv files (Supplementary File 2) for ML analysis using Python programming language (v3.7.6) and Scikit-learn package (v0.22.1) in Jupyter NoteBook (v6.0.3). Then, MSPs were grouped by their AMR profiles and eight distinct files have been created according their AMR classes and susceptibility, i.e., S, Cip*^R^*, Tet*^R^*, Amp*^R^*, Ery*^R^*, Gent*^R^*, Strep*^R^*, and Kana*^R^* (Figure 1). Category names (e.g., S and R) were binarized, where 0 and 1 represented MSPs susceptible and resistant to the AMR class studied, respectively. All peaks, here called features, were transformed using a Min-Max scaler which transformed values into the (0,1) range. Such a step is necessary to bring different variables at the same level, as variables that are measured at different scales may not contribute equally to the model fitting.

#### Feature’s Selection

Dataset with many features, which could be redundant or irrelevant, may lead to an overcomplicated algorithm with low prediction accuracy and long training time. Feature selection is the process of choosing relevant features, to use in a classification model construction, either to improve accuracy scores or to boost performance. For this purpose, a meta-transformer based on a Random Forest estimator, implemented into scikit-learn library, was used to discard irrelevant features.

#### Model Selection

MSPs were randomly split into 70% training and 30% test datasets, with a stratification based on their binarized AMR profiles. The training dataset is implemented to build up a prediction model, while the test dataset is used as an external validation step of the trained model. For each studied AMR classes, Random Forests (RF), Logistic Regression (LR), and Naïve Bayes (NB) models were built, as they are common algorithms used in microbiology (Goodswen et al., 2021). RF is currently among the most used ML methods due to its robustness. It is essentially a collection of independent decision trees, where each tree could be different from the others, as the algorithm will make completely different random choices to make sure trees are distinct. Such algorithms make aggregated predictions using a group of decision trees. LR is a linear classifier, which predicts the probabilities of success and failure event. It is easy to implement and interpret and efficient to train. NB classifier assumes that the presence of a particular feature is not related to the presence of another feature. It is easy to interpret and is often applied for many medical applications. The area under the precision recall curve (AUPRC) was investigated to determine the most performant model (data not shown).

#### Tuning

Upon selection of the best performing model, it was optimized by looking for the best combination of hyper-parameters according to the F1-score, described in the metrics section. Hyper-parameters for each selected model were tuned by using an instance which generates candidates from a grid of given parameter values, a grid search, with a 10-fold cross validation, with a scoring method looking for the more optimized F1-score. K-fold cross validation is a resampling method, which estimates the performance of the ML model.

The 0.5 default probability score threshold may not represent an optimal interpretation and can result in poor performance. Therefore, a threshold adjustment was investigated to bring a higher predictive performance (Weis et al., 2020a). A threshold selection, for each classifier, based on their precision recall curve (PRC) was applied, according to the best F1-score. In the case of imbalance classes, like the current dataset, PRC can suggest an optimal threshold (Saito and Rehmsmeier, 2015). In this study, detection of resistant isolates (true positives) is the key point of the study. PRC is based on true positive values, i.e., true positive and positive predictive values, among positive prediction. Hence, PRC relies on positive classes regardless of true negative value, making it a tool of choice for the study threshold selection. In the end, values less than the custom threshold are assigned to class 0, or susceptible, while value greater than or equal to the custom threshold are assigned to class 1, or resistant.

#### Performance and Metrics

As a next step, performance of the selected classifier needed to be assessed on data not yet seen by the model. For this, an external validation has been carried out by using the test dataset. Classification of spectra was summarized in a confusion matrix. From it, several performance metrics, such as the specificity, the recall, the precision or the positive predictive value (PPV), the negative predictive value (NPV), and area under the receiver operating characteristic curve (AUROC) and PRC were calculated. The PPV tells us how much we can trust the model when a resistant result is predicted, and in the other way, the NPV tells us how much we can trust the model when a sensitive result is predicted. The recall, also called sensitivity, measures how the model can find all positive units. The specificity refers to the model’s ability to give a negative result when an isolate is susceptible. The ROC curve is a graphical way to represent the performance of the classifier for all threshold classifications, with the false-positive rate and true-positive rate as axis. Therefore, the AUROC can be used to measure the model’s discriminative ability. Usually, an AUC of 0.5 is assimilated to a non-discriminative model, while 0.7–0.8 is considered acceptable, 0.8–0.9 is excellent, and more than 0.9 is considered outstanding (Hosmer et al., 2013). Along the same line, the PRC is a graphical visualization that combines the precision and the recall. The higher curve on the *y*-axis, the better the performance. Therefore, the AUPRC returns a value between 0 and 1, where 0 is the worst and 1 is the best. Finally, the F1-score is calculated from the precision and the recall. It conveys balance between the precision (PPV) and the recall (sensitivity).

Detailed information on ML analysis is shown in Supplementary File 3.

#### Biomarker Identification

Features of importance, based on RF algorithm trained on the whole dataset, were investigated to potentially identify already known antimicrobial resistance mechanisms or new antimicrobial targets. It rates how important each feature is for the decision tree. A score based on between 0 and 1 for each feature is calculated, where 0 means ‘‘Not used’’ and 1 highlighted a ‘‘perfect biomarker.’’ Score for features of importance is computed as the mean and standard deviation of accumulation of the impurity decrease within each tree. Therefore, it describes the relevancy of a peak and, hence, can help to understand the biological problem. The five first features with the higher importance were checked in on Uniprot^1^ according their mass in Da. Average theoretical masses were calculated using the online Expasy portal tool^2^ based on Uniprot amino acid sequence.

#### Statistical Analysis

Effects of extraction methods on AMR predictions were analyzed based on analysis of variance (ANOVA) of the sum of AUPRCs of the different antimicrobial classifiers. ANOVA assumptions were verified with a Shapiro-Wilks and Levene tests. Shapiro-Wilks test determines if your data are normally distributed. The Leven test evaluates the equality of the variance. Differences were considered significant at *p* < 0.05.

## Results

### Spectra Quality and Reproducibility

**FIGURE 2:**
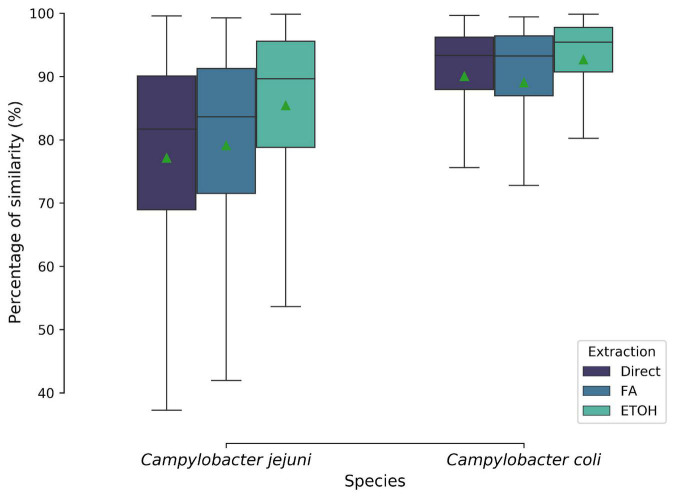
Biological reproducibility of MALDI-TOF mass spectra based on their protein extraction type and species level. Boxplots show the isolates average similarities in percentage. Green triangle represented the mean. Direct, direct deposit; FA, formic acid extraction; EtOH, ethanol/acetonitrile extraction.

A total of 9,180 mass spectra were generated. An average identification log score of 2.0 was obtained for all spectra. Outlines, flatlines, and spectra not identified at the *Campylobacter* genus level were discarded for the analysis, resulting into 9,173 spectra. The latter was transformed into 1,020 MSPs, including 672 and 348 MSPs for *C. jejuni* and *C. coli*, respectively. Three different types of extractions, i.e., off-plate ethanol/acetonitrile extraction, direct deposit, and on-plate acid formic extraction, were carried out for both species. Hence, reproducibility was tested for the three biological replicates. Average similarities in percentage between the type of extraction and species are provided in Figure 2. For both species, no significant differences were observed between off- and on-plate extractions. Average similarity of means ranged from 77.1 to 92.7% between biological replicates for *C. jejuni* and *C. coli*, respectively.

### Antimicrobial-Specific Screening

**FIGURE 3:**
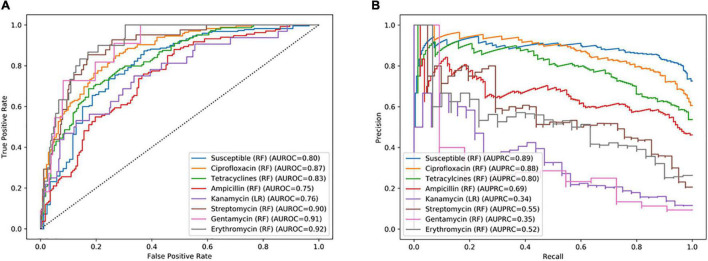
**(A)** Receiver operating characteristic (ROC) curve and **(B)** recall–precision (PR) curves, and their related area under the curve, of specific antimicrobials based on combined *C. jejuni* and *C. coli* MALDI-TOF main protein spectra profiles (MSPs) of the test set (30%, *n* = 306). RF, Random Forest algorithm; LR, Logistic Regression algorithm; AUROC, Area Under the ROC Curve; AUPRC, Area Under the Precision Recall Curve.

As a first step, different ML models, i.e., RF, LR, and NB, were trained for specific antimicrobials from different classes, regardless of the species identification to evaluate the potential of fast AM-screening without knowing the microbial identification. For this purpose, 1,020 MSPs, combining the three types of extractions and the two species, were split into a training and a validation set. The training set served to build the model, and the test set, to evaluate the performance of the model. Seven classifiers were built with RF and one with an LR algorithm. ROC and PR curves were computed to investigate the model’s performance for each antibiotic (Figure 3), as well as other evaluation metrics such as sensitivity, specificity, PPV, and NPV summarized in Table 2.

**TABLE 2:** Performance of retained machine learning classifier using combined *C. jejuni* and *C. coli* MALDI-TOF main protein spectra profiles (MSPs) of the test set (30%, *n* = 306 MSPs), grouped by the resistance profile.

Species	Antibiotics	Sensitivity (%)	Specificity (%)	PPV (%)	NPV (%)
*C. jejuni* and *C. coli* (*n* = 306 MSPs)	Susceptible* (*n* = 86)	92.3	45.3	81.2	69.6
	Ciprofloxacin (*n* = 165)	90.9	63.8	74.6	85.7
	Erythromycin (*n* = 30)	80.0	88.4	42.8	97.6
	Tetracycline (*n* = 144)	87.5	62.3	67.4	84.9
	Ampicillin (*n* = 133)	90.2	47.4	56.9	86.3
	Kanamycin (*n* = 32)	43.8	91.6	37.8	93.3
	Streptomycin (*n* = 41)	78.0	87.2	48.5	96.3
	Gentamycin (*n* = 11)	72.7	93.6	29.6	98.9

Threshold applied for metrics calculation is based on the best F1-scores. PPV, positive predictive value; NPV, negative predictive value. *Susceptible to all tested antimicrobials.

Among the eight antimicrobials tested, three models performed better than the other considering both AUROC and AUPR curves. The best-performing model was the classifier allowing the distinction between resistant and completely susceptible isolates, with an area of 0.80 and 0.89 under the ROC and PR curves, respectively. The ciprofloxacin and tetracycline classifiers were the two other performant models according to their AUROC and the AUPR curves, an area of 0.87, 0.83, and 0.88, 0.80 under the AUROC and AUPRC, respectively (Figure 3). While the specificity was low for the three models, with a maximum of 63.8%, a sensitivity ranging from 87.5 and 92.3% was obtained (Table 2). Additionally, 74.6 and 85.7% of predicted values of the ciprofloxacin classifier could be reliable for resistant and susceptible values, respectively.

Remaining models had an AUROC of up to 0.92. However, considering the precision and the recall, they performed poorly. Indeed, the AUPR curve was between 0.34 and 0.69. Sensitivity and specificity may be high, but PPVs were low, e.g., 80.0, 88.4, and 42.8%, respectively, for the erythromycin model.

### Species-Specific Screening

**FIGURE 4:**
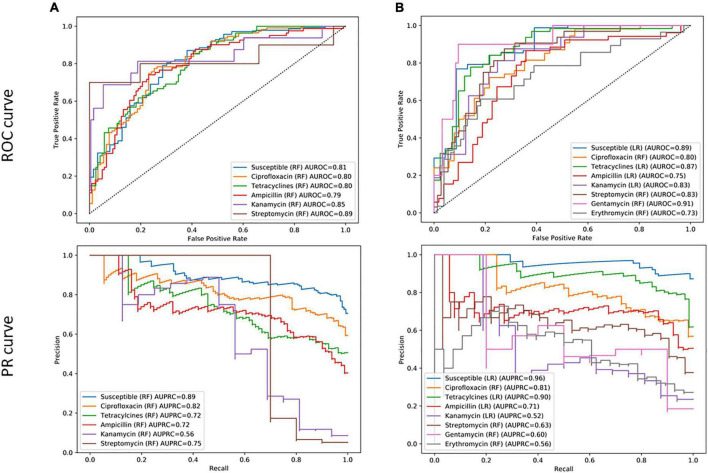
Receiver operating characteristic (ROC) curve and recall–precision (PR) curves, and their related area under the curve, of specific antimicrobials based on 202 *C. jejuni* **(A)** and 105 *C. coli* **(B)** MALDI-TOF main protein spectra profiles (MSPs) of the test set (30%). RF, Random Forest algorithm; LR, Logistic regression algorithm; AUROC, Area under the ROC curve; AUPRC, Area under the precision–recall curve.

In a second phase, *C. coli* and *C. jejuni* MSPs were investigated separately to look over potential differences between tested antimicrobials. Previously, ROC and PR curves and their respective area under the curve have been computed, based on 202 and 105 MSPs, for the *C. jejuni* and *C. coli* test sets, respectively (Figure 4). As well, performance metrics were calculated (Table 3). Due to few gentamycin- and erythromycin-resistant isolates for *C. jejuni* in the initial collection (one and two, respectively), no model was built for these two antibiotics. RF and LR were once again fitting the best data. All six *C. jejuni* models were based on RF algorithms. Four models were built using LR and the remaining four were built using RF algorithms for *C. coli*.

**TABLE 3:** Performance of retained machine learning classifier using *C. jejuni (n* = 202 MSPs) and *C. coli* (*n* = 105 MSPs) MALDI-TOF main protein spectra profiles (MSPs) of the test set (30%), grouped by the resistance profile.

Species	Antibiotics	Sensitivity (%)	Specificity (%)	PPV (%)	NPV (%)
*C. jejuni* (*n* = 202 MSPs)	Susceptible* (*n* = 63)	92.8	55.6	82.2	77.8
	Ciprofloxacin (*n* = 111)	96.4	41.8	66.9	90.5
	Erythromycin (*n* = 2)	NA	NA	NA	NA
	Tetracycline (*n* = 81)	92.6	47.1	53.9	90.5
	Ampicillin (*n* = 81)	77.7	70.3	63.6	82.5
	Kanamycin (*n* = 16)	62.5	97.9	71.4	96.8
	Streptomycin (*n* = 10)	70.0	100.0	100.0	98.5
	Gentamycin (*n* = 1)	NA	NA	NA	NA
*C. coli* (*n* = 105 MSPs)	Susceptible* (*n* = 23)	98.8	60.9	90.0	93.3
	Ciprofloxacin (*n* = 54)	98.2	45.1	65.4	95.8
	Erythromycin (*n* = 28)	71.4	70.1	46.5	87.1
	Tetracycline (*n* = 63)	96.8	61.9	79.2	92.9
	Ampicillin (*n* = 52)	86.5	64.1	70.3	82.9
	Kanamycin (*n* = 16)	62.5	86.5	45.5	92.7
	Streptomycin (*n* = 32)	84.3	75.3	60.0	91.7
	Gentamycin (*n* = 10)	70.0	93.7	53.8	96.7

Threshold applied for metrics calculation is based on the best F1-scores. PPV, positive predictive value; NPV, negative predictive value. *Susceptible to all tested antimicrobials. NA, Not applicable due to few isolates in the category.

As described in the specific antimicrobial section, the susceptible, ciprofloxacin, and tetracycline classifiers were the three best-performing models in both species, with an AUROC and AURP curve ranging from 0.80 to 0.89 and from 0.72 to 0.96, respectively (Figure 4). The susceptible classifier was the more performant model in both *C. jejuni* and *C. coli*. Tetracycline classifier was the second more effective model for *C. coli*, with an AUROC of 0.87 and AUPRC of 0.90, while it was the ciprofloxacin classifier for *C. jejuni*, with an AUROC of 0.80 and AUPRC of 0.82. Overall, sensitivity values up to 98.8% were obtained for these models. High PPVs and NPVs were obtained for susceptible classifiers. *C. coli* tetracycline classifier also performed well with a 79.2 and 92.9% for PPV and NPV, respectively. Surprisingly, the ciprofloxacin classifier was less efficient in both species. Indeed, a lower PPV was obtained, i.e., 10% differences, in comparison with previous results where the microbial identification was not taken into consideration. For erythromycin, kanamycin, and gentamycin classifiers, observations described in the previous section could be assessed.

Differences were observed for the ampicillin and streptomycin classifier for *C. coli* and *C. jejuni*. *C. jejuni* streptomycin’s classifier performed more efficiently than the one of *C. coli*. PPVs and NPVs of 100 and 98.5%, against 60.0 and 91.7%, were calculated, respectively. *C. coli* ampicillin’s classifier was more performant than that of *C. jejuni*, while similar AUROC and AUPR curves were found. Indeed, PPVs and NPVs of 70.3 and 82.9% against 63.6 and 82.5% were calculated for *C. coli* and *C. jejuni*, respectively (Table 3).

### Protein Extraction Impact on Resistance Predictions

**FIGURE 5:**
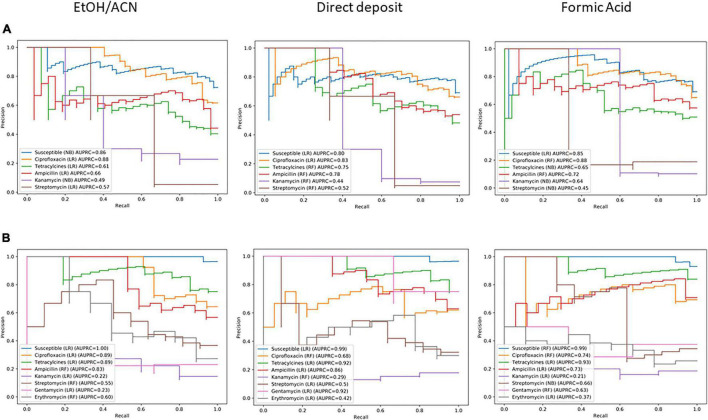
Comparison of precision-recall curves for the three-extraction tested on **(A)** *C. jejuni* (*n* = 68 MSPs) and **(B)** *C. coli* (*n* = 35 MSPs) of the test set (30%). EtOH/ACN: complete ethanol/acetonitrile-based proteins extraction. RF, Random Forest algorithm; LR, Logistic Regression algorithm; NB, Nave Bayes algorithm.

Thirdly, methods of extraction, i.e., direct deposit, FA on-plate, and EtOH/ACN off-plate extraction, were investigated to check potential variation for specific antimicrobials. Thereby, MSPs acquired for each extraction for both *C. jejuni* (*n* = 224 MSPs) and *C. coli* (*n* = 116 MSPs) were used to build a specific ML model per antimicrobial. Models are compared in Figure 5. The ANOVA resulted in 0.976 and 0.936 (*p* > 0.05) values for *C. jejuni* and *C. coli*, respectively. Therefore, the null hypothesis, i.e., there is no difference between extraction methods, is retained.

**FIGURE 6:**
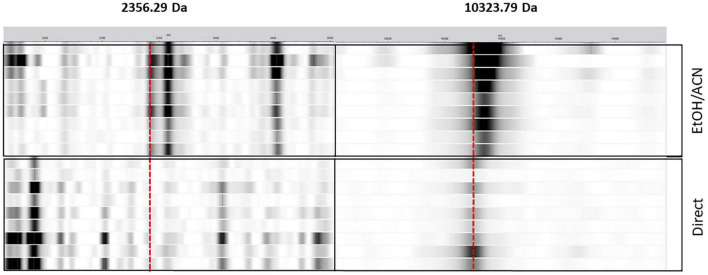
Pseudogel view representation of mass spectra from *C. coli* from the direct deposit (direct, *n* = 8) and the ethanol/acetonitrile off-plate extraction (EtOH/ACN, *n* = 8). The *x*-axis represents the mass-to-charge (m/z) ratio in Da. Strips intensities is function of the peak intensity. The red dashed lines represent the observed peaks, i.e., 2,356.29 and 10,323.79 Da.

Nevertheless, in the case of the *C. coli* gentamycin’s classifier, while the performance is low for the EtOH/ACN extraction (AUPRC = 0.23), the classifier for the direct deposit is more efficient (AUPRC = 0.92). Features of extractions for both classifiers were investigated. For the EtOH/ACN classifier, 2,356.29 Da was the more important feature. For the direct deposit classifier, 10,323.79 Da was the more important feature. While these features in a model were particularly important, they were the less important features in the other model. The 10,323.79 Da peak was detected in both extractions, while softly shifting for the EtOH/ACN, i.e., 10,333.67 Da. The 2,356.29 peak was not detected in the direct deposit (Figure 6).

### Biomarkers: Antimicrobial Resistance Mechanisms

RF classifiers performing the best, i.e., susceptible, ciprofloxacin, and tetracycline, while microbial species is not known, were used to retrieve features of importance. Then, the Uniprot database was investigated to potentially identify each feature according their mass in Dalton, regardless post-translational modifications. Table 4 summarizes the top five features for each classifier. When several proteins had the same mass, proteins with the most probable function linked to AMR were retained. No protein for *C. jejuni* or *C. coli* was identified at 6,436.22 Da. The DNA methyltransferase at 6,436 Da was in *Helicobacter pylori*, a closely related genus of *Campylobacter*.

**TABLE 4:** Top five ranking of Random Forest features of importance.

Classifier	Rank	Features (Da)	Average theoretical mass (Da)	Protein	UniProt ID
**Susceptible**	1	8460.76	8460.07	Transcriptional regulator	A0A1T1ZLP8
	2	3257.41	3256.98	GNAT family N-acetyltransferase	A0A6N3Q833
	3	5867.81	5867.86	ATP-binding protein	A0A2A5MAC7
	4	2766.98	2767.13	Poly(A) polymerase	A0A5T1K937
	5	4365.25	4364.39	50 S ribosomal protein L36	A0A1E7P1M9
**Ciprofloxacin**	1	6436.22	6435.55	DNA methyltransferase*	A0A438RVN3*
	2	2766.98	2767.13	Poly(A) polymerase	A0A5T1K937
	3	2241.84	2241.67	Type II toxin-antitoxin system HicB family antitoxin	A0A691V648
	4	3257.41	3256.98	GNAT family N-acetyltransferase	A0A6N3Q833
	5	7083.30	7083.03	MmgE/PrpD family protein	A0A4Y8C2R1
**Tetracycline**	1	4365.25	4364.39	50 S ribosomal protein L36	A0A1E7P1M9
	2	2766.98	2767.13	Poly(A) polymerase	A0A5T1K937
	3	7083.30	7083.03	MmgE/PrpD family protein	A0A4Y8C2R1
	4	6436.22	6435.55	DNA methyltransferase*	A0A438RVN3*
	5	2713.95	2713.06	Superoxide dismutase	A0A431FY74

Da, Dalton. *Identified in the closely related genus Helicobacter pylori (former Campylobacter pylori).

## Discussion

Several reports described MALDI-TOF MS as a more time- and cost-effective alternative approach to current classic AST methods (Hrabák et al., 2013; Oviaño and Bou, 2019). Being combined with ML, such an approach may be even more relevant for AST in routine diagnostics (Weis et al., 2020b). However, to our knowledge, no study implying relevant foodborne pathogens for AMR screening has been published yet. Therefore, the scope of this study was to consider whether a mass spectrometry technique combined with an ML approach could be utilized for a combined rapid species identification and AMR screening for foodborne pathogens.

The main result of this study was to observe whether mass spectra with 91 protein peaks selected by automatic peak-matching could predict with a high average sensitivity and precision the strains’ susceptibility and resistance to ciprofloxacin and tetracycline, independent of the microbial species identification. Therefore, these models were missing very few resistant isolates. Similarly, Weis and colleagues, computed an AUROC for 42 different antibiotics on a large “real-world” clinical dataset by combining multiple species (Weis et al., 2020a). They pointed out that they reached AUROC values above 0.90 for 23 of the tested antibiotics. Such results support the idea that mass spectra could provide far more than simple species information. Nevertheless, in the literature, most of the publications focused on specific species such as *S. aureus*, *Escherichia coli*, and *Klebsiella pneumoniae*. Additionally, they mainly analyzed one type of antimicrobial classes, e.g., glycopeptides such as vancomycin (Mather et al., 2016; Asakura et al., 2018; Wang et al., 2018; Candela et al., 2021). For example, Asakura et al. (2018) obtained a sensitivity of 99.0% and a specificity of 88.0% while comparing vancomycin-susceptible and heterogeneous vancomycin intermediately resistant *S*. *aureus.* Wang et al. (2018) obtained similar results with a 77.0 and 81.4% sensitivity and specificity, respectively, for the same comparison. When comparing *C. jejuni* and *C. coli* separately and for different antimicrobials, we found that susceptible, ciprofloxacin, and tetracycline classifiers were the three best-performing models in both species, while the others performed less accurately. Similarly to other studies, a sensitivity ranging from 92.6 to 98.8% was obtained for both species and the three performant classifiers. Weis et al. (2020a) also looked at species-specific antimicrobial resistance prediction for *S. aureus*, *E. coli*, and *K. pneumoniae*. They reported an AUROC ranging from 0.77 to 0.81, and an AUPRC ranging from 0.52 to 0.70 for ciprofloxacin predictions. In the current study, similar AUROC values were found but a higher AUPRC was observed with 0.82 and 0.81 for *C. jejuni* and *C. coli*, respectively, meaning that the current model may accurately predict ciprofloxacin-resistant isolates. Considered as a critically important antimicrobial, ciprofloxacin is widely used for the treatment of broad human bacterial infections, including enteric ones (WHO, 2019). Therefore, early screening of its resistance may play an essential role for the administration of the definitive antimicrobial therapy. Nevertheless, the comparison between the different studies is intricate to perform due to the number of isolates, the genus analyzed, the type of extraction, as well as the type of algorithm used. In the current study, classifiers performing poorly, i.e., kanamycin, streptomycin, gentamycin, and erythromycin, were subject to a highly imbalanced dataset, with an average of 10/90 resistant/susceptible ratio, instead of a close 50/50 ratio one (e.g., 36 gentamycin-resistant MSPs for 984 gentamycin-susceptible MSPs). Precision disparities were observed for the ciprofloxacin, ampicillin, and streptomycin classifiers of both species, in comparison to classifiers not considering the species level. While such differences could be attributed to the unbalanced number of resistant isolates for ampicillin and streptomycin, the ciprofloxacin classifier was in contrast well balanced. The ciprofloxacin classifier may be less effective for predictions, while looking specifically at the species level. In the end, prediction based on protein mass spectra grouped by AMR, regardless of bacterial species, may be the best option for an efficient and swift AMR-screening. Such observations might also be explained by average similarity differences obtained between *C. jejuni* and *C. coli*. Cuénod and Egli (2021), Cuénod et al. (2021) reported that the preparation protocol used, the duration of incubation, maintenance of the device, for example, could potentially impact the quality of the spectra. Inevitably it may have influenced the final prediction for both species. Hypothetically, such observations may also show that AMR screening by MALDI-TOF MS is going beyond the bacterial genus or species and might be directly linked to the resistance mechanism and protein/metabolite expression itself. To our knowledge, this is the first study establishing that ML and MALDI-TOF MS could be applied for AMR screening of foodborne pathogens, such as *Campylobacter* spp.

Nevertheless, in the current study, the specificity was not as high as the specificity described by the previously mentioned studies. While creating the ML pipeline, sensitivity was chosen as the most important parameter to adjust the threshold score during the tuning part. Hence, the optimal threshold was selected based on the F1-score, meaning the best compromise between higher sensitivity and precision, specific to each classifier. Classifiers guiding antibiotic therapy decision must have high sensitivity (Weis et al., 2020a). On the one hand, assuming an isolate is susceptible, while it is resistant, may lead to an ineffective treatment and eventually have an important impact on patient management. On the other hand, assuming an isolate is resistant, while it is susceptible, may still lead to an effective treatment. However, while seeking and picking to have high sensitivity, it will inevitably decrease the specificity, by decreasing it. In the previously cited reports, threshold adjustments were not mentioned. Therefore, threshold adjustment may be a key step while elaborating ML pipeline for routine laboratories based on MALDI-TOF mass spectra.

The impact of protein extraction methods was also evaluated. Indeed, the EtOH/ACN extraction is the most popular extraction protocol when it comes to research investigations. However, the direct deposit and the on-plate FA extractions are the most straightforward methods used in routine laboratories. No significant differences were observed between the direct deposit, the FA on-plate, and the EtOH/ACN extraction. Therefore, in order to rapidly obtain straightforward AMR assessment information, the application of the direct deposit method could be applied for species identification as well as AMR screening in *Campylobacter*. Interestingly, *C. coli* gentamycin classifier performance was different between EtOH/ACN extraction and the direct deposit. Indeed, with a simple biological smear on the MALDI-TOF target, gentamycin’s prediction was more precise. Surprisingly, the absence of the 2,356.29 Da peak resulted in a higher AUPRC for the direct deposit classifier. In the literature, the loss of a specific peak between different types have already been described (Josten et al., 2014). However, in their case, the loss of a protein happened during the ethanol washing step of the EtOH/ACN extraction. Thus, the peptide was only present during a direct deposit measurement. However, to confirm our observation, additional gentamycin-resistant isolates should be analyzed as currently too few gentamycin isolates are present in the current dataset.

Along the same line, putative biomarkers have been identified for each class of studied antibiotics by looking into RF algorithm features of importance. Majority of these proteins, such as transcriptional regulator, ATP-binding, GCN5-related N-acetyltransferase, DNA-methyltransferase, toxin-antitoxin system, PrpD, and superoxide dismutase proteins had a direct or indirect link with already known antibiotic resistance, tolerance, or spread mechanisms in different genera of bacteria (e.g., *Salmonella*, *Enterococcus*, *Escherichia*, *Mycobacterium*, and *Pseudomonas*) (Draker and Wright, 2004; Yugendran and Harish, 2016; Hicks et al., 2018; Kang et al., 2018; Martins et al., 2018; Su et al., 2018; Shaheen et al., 2020). Nevertheless, *Campylobacter*’s AMR mechanisms are either chromosomal mutations, such as the single mutation C257T in the *gyrA* gene or the A207G mutation in the 23 S rRNA gene for ciprofloxacin and erythromycin, respectively, or acquired genes, such as *tet(O*), *bla_*OXA*_-61* and *aph(3’)-III* for tetracycline, ampicillin, and gentamycin resistances, respectively (Payot et al., 2006; Iovine, 2013). Overall, these mechanisms are working in synergy with the *cmeABC* efflux pump or porines, such the Major-Out-Membrane Porines (MOMP) (Lin et al., 2002). Over the biomarkers identified as relevant by RF susceptible classifier, the GCN5-related N-acetyltransferase and the 50 S ribosomal protein L36 may be linked to already known aminoglycosides or tetracyclines resistance mechanisms of *Campylobacter*, respectively. On one hand, aminoglycoside-modifying enzymes, such as acetyltransferase [e.g., *aac(6′)-Ie*–*aph(2″)-If2]* were already detected in gentamycin-resistant *Campylobacter* isolates (Zhao et al., 2016). On the other hand, the Tet(O) ribosomal protection protein is known to bind on both 30S and 50S subunits, conferring tetracycline resistance (Li et al., 2013). Interestingly, the L36 proteins were the first feature of importance highlighted for the tetracycline classifier. Identification of specific proteins directly implied to AMR mechanisms, while using MALDI-TOF MS within the 2–20 kDa range, could be problematic (Welker and Van Belkum, 2019). Indeed, proteins responsible for resistances are large proteins (e.g., GyrA = 96,974 Da). Therefore, in case an indicative biomarker is identified, it may not be a necessary protein conferring the resistance itself, but it may be a protein or peptide co-coded on the plasmid of the protein responsible of the resistance (Lau et al., 2014). Therefore, the 4,365.25 m/z peak may be a biosignature linked to the presence of the *tet(O)* gene. In the literature, two protein biomarkers, i.e., 3,665.79 *m/z* and 6,036.59 *m/z*, have been reported to be a potential biomarker of the tetracycline resistance in other bacterial genera (Sabença et al., 2020; Sousa et al., 2020). However, these biomarkers were not observed here. Along the same line, the 6,436.22 Da protein was considered as the most important feature for the ciprofloxacin’s classifier. The protein was identified as a DNA methylase in *H. pylori*, formerly related to the *Campylobacter* genus. Yugendran and Harish put in light the hypothesis that ciprofloxacin-resistance in *E. coli* may be induced by DNA methylation, leading to the possible involvement of some mechanism other than the quinolone-resistance determining region (QRDR) capable of inducing fluoroquinolone resistance (Yugendran and Harish, 2016). While the single point mutation in *gyrA* represents the major fluoroquinolones resistance mechanism in *Campylobacter*, such venue may be worth exploring in the future. Other potential ciprofloxacin biomarkers, neighboring 6,300 Da, were put recently in light for other *E. coli* (Sousa et al., 2020) and *Enterococcus* (Sabença et al., 2020; Sousa et al., 2020). Nevertheless, interpretation on the biological role of features may be cautiously interpreted, and a peptide sequencing by tandem mass spectrometry should be performed to assess the real biological function of these biomarkers.

Little is known on the impact of such approaches as described here on the health management potential cost savings in clinical practice. Weis and colleagues affirmed in their study that the application of such workflow provided a treatment guidance 12–72 h earlier than classical approaches and to have a significant impact on the physician–patient workflow (Weis et al., 2020a). It is worth mentioning that the ML is intended for supporting the decision making process. Therefore, it is a support giving guidance on possible resistance outcomes that lead early antibiotherapy in a specific direction. ML may be used as an AMR screening tool, displaying an alert message on the MALDI-TOF MS microbial identification report, when the isolate is classified as a positive category value. It is already the case for several Bruker subtyping modules (e.g., MRSA, cfiA positive or bla_*KPC*_ modules). Therefore, instead of giving an empirical treatment until the AMR confirmation by reference AST, the patient’s antibiotherapy may be defined faster (e.g., 24 h earlier).

Phenotypic antibiogram should still follow up to establish the AMR profile and, in case, reorient the antibiotherapy. Additionally, 2025 AMR monitoring of food-producing isolates, such as ESBL/AmpC/carbapenemase-producing *E. coli*, will be done by WGS (Aerts et al., 2019). Therefore, a combination of MALDI-TOF MS, ML, and WGS could be an interesting monitoring tool with a relevant impact on the control of the emergence of AMR in the European Union. As well, the application of MALDI-TOF MS in microbiology for lipid investigation has conceptualized several breakthroughs for AMR screening (Bruker, 2019; Furniss et al., 2019; Dortet et al., 2020). In case of the ability of such method to distinct microbial lipids directly from body fluids such as serum, blood, and urine, there will be no need of a culture step (Solntceva et al., 2021). So far, only the last-line treatment for multidrug-resistant Gram-negative bacteria, i.e., polymyxin, has been investigated without a ML approach. Lipidomics combined to artificial intelligence may be a new venue to explore AMR problem cases that proteomics could not solve. However, there is still a stony way before the long-term implementation of ML in routine laboratories for AMR screening. Nevertheless, a single protein mass spectra may be used in the future as an utmost “One-fits all” diagnostics tool for: species identification, AMR screening, and genetic diversity (Feucherolles et al., 2021).

Several limitations of our study are offered for consideration. First, the employed dataset might be considered as relatively small to train an ML algorithm properly. Indeed, lack of data could lead a model to overfit or underfit the data. Several models (e.g., gentamycin or kanamycin) were trained on heavy unbalanced classes, which is not recommended to build a robust and reliable tool for AMR predictions. Therefore, extra isolates resistant to these antimicrobials should be added to the current dataset. Additionally, only three ML algorithms, i.e., RF, LR, and NB, were tested. The support vector machine algorithm was not included in the study, while it is also a widely used algorithm for AMR predictions. Another limitation of the study is the use of disk-diffusion antibiograms, which—while being a valid and highly reproducible method to characterize an isolate as resistant or susceptible—do not allow quantifying the minimal inhibitory concentration (MIC) of a given antibiotic. Additionally, it would have been possible to test for further antibiotics, e.g., carbapenems. The final limitation of this study could be the fact that the RF model, used for putative biomarkers identification, was trained on the whole dataset. Indeed, under these settings, there is no proof that these biomarkers could work in a given analysis. For such investigations, the model should have been trained on a split dataset, including a training and test set, with a 70/30% ratio, respectively.

## Conclusion

On the one hand, MALDI-TOF MS in combination with supervised ML may be a powerful tool for the fast screening of foodborne pathogens such as *C. coli* and *C. jejuni*, which might be susceptible, ciprofloxacin, or tetracycline resistant. On the other hand, other antimicrobials tested, i.e., ampicillin, gentamycin, kanamycin, streptomycin, and erythromycin, did not provide good results to reach a conclusion for its application under clinical settings, due to unbalance datasets. Nonetheless, this work could serve as a proof-of-concept, and future research should include other important foodborne pathogens such as *Salmonella* spp. Our approach has the potential to obtain the following information from one single protein spectrum analysis: species identification, antimicrobial susceptibility patterns, and genetic diversity.

## Data Availability Statement

The original contributions presented in the study are included in the article/Supplementary Material, further inquiries can be directed to the corresponding author/s.

## Author Contributions

MF carried out MALDI-TOF MS, machine learning, and data analysis, and drafted the manuscript with MN and CR. MN and CR isolated and performed the identification and AMR characterization of the *Campylobacter* collection. SB and DM supplied extra *Campylobacter* strains from their respective clinical laboratories. CR, SB, and DM provided their expert critical point of view on the current work. SL gave access to his lab for all mass spectrometry analysis. H-MC supervised the project. CP wrote the project proposal and obtained funding. All authors contributed to the formal analysis, writing, review, and editing of the manuscript, read and agreed to the published version of the manuscript.

## Conflict of Interest

The authors declare that the research was conducted in the absence of any commercial or financial relationships that could be construed as a potential conflict of interest.

## Publisher’s Note

All claims expressed in this article are solely those of the authors and do not necessarily represent those of their affiliated organizations, or those of the publisher, the editors and the reviewers. Any product that may be evaluated in this article, or claim that may be made by its manufacturer, is not guaranteed or endorsed by the publisher.

## Footnotes

1
https://www.uniprot.org/

2
http://web.expasy.org/compute_pi/

## Supplementary Material

Supplementary File 1 Details of the *Campylobacter* spp. collection used for the study.

Supplementary File 2 *C. jejuni* and C. coli peak matching table for the EtOH/ACN, FA extraction, and direct deposit.

Supplementary File 3 Example of one of the supervised machine learning Python workflows.
