# Supplementary material for: Combination of MALDI-TOF Mass Spectrometry and Machine Learning for Rapid Antimicrobial Resistance Screening: The Case of Campylobacter spp
Source: Front Microbiol. 2022 Feb 18;12:804484. doi: 10.3389/fmicb.2021.804484 (PMC8894766; doi:10.3389/fmicb.2021.804484)
Supplement: Supplementary file 2 [file Data_Sheet_2.PDF]

```
#!/usr/bin/env python
# coding: utf-8

# In[1]:
#Import
import pandas as pd
import matplotlib.pyplot as plt
import numpy as np
import scipy as sp
import IPython
import sklearn

# In[2]:
#Import Dataset
jejuni = pd.read_csv ('Peak_matching_table_Ccoli_Cjejuni_Penny_Character_Mixed_IC_Cip.csv')
print(jejuni.groupby('12').size())
Y = jejuni['12']
X = jejuni.drop(columns=['12'])

# In[3]:
#Features transformation/Scaling
from sklearn.preprocessing import MinMaxScaler
scaler = MinMaxScaler()
jejuni_scaled = scaler.fit_transform(X)
print('Scaled dataframe',jejuni_scaled)

# In[4]:
#Save the new dataframe
jejuni_scaled_df= pd.DataFrame(jejuni_scaled, Y)
jejuni_scaled_df
pd.DataFrame(jejuni_scaled, Y).to_csv('jejuni_scaled_df_RS.csv')
```

```
#New variables
jejuni_2 = pd.read_csv ('jejuni_scaled_df_RS.csv')
print(jejuni.groupby('12').size())
Y = jejuni_2['12']
X = jejuni_2.drop(columns=['12'])

# In[5]:
#Features selection
from sklearn.ensemble import RandomForestClassifier
from sklearn.feature_selection import SelectFromModel
from sklearn.model_selection import cross_val_score
clf = RandomForestClassifier(random_state=0)
clf = clf.fit(X, Y)
clf.feature_importances_
model = SelectFromModel(clf, prefit=True)
X_new = model.transform(X)

#Save the new dataframe
jejuni_scaled_Features_df= pd.DataFrame(X_new, Y)
jejuni_scaled_Features_df
pd.DataFrame(jejuni_scaled_Features_df, Y).to_csv('coli_scaled_Features_df_Cip.csv')
print('Avant:',X.shape)
print('Après:',X_new.shape)
features = X.columns.values
print(features[model.get_support()])


# In[6]:
#Training/Validation/Test set
from sklearn.model_selection import train_test_split
```

```
X_train, X_test, Y_train, Y_test = train_test_split(X_new, Y, test_size=0.3,stratify=Y,random_state=0,
shuffle=True)

print('X_test:', X_test)

print ('X_test shape', X_test.shape)


# In[7]:

#Model selection

from sklearn.metrics import plot_roc_curve, roc_curve, auc, precision_recall_curve,
plot_precision_recall_curve, roc_auc_score

from sklearn.ensemble import RandomForestClassifier

from sklearn.linear_model import LogisticRegression

from sklearn.naive_bayes import GaussianNB

LR = LogisticRegression().fit(X_train, Y_train)

NB = GaussianNB().fit (X_train, Y_train)

rf = RandomForestClassifier().fit(X_train,Y_train)


#ROC Curve

classifiers = [LR, NB, rf]

ax = plt.gca()

for i in classifiers:

   plot_roc_curve(i, X_test, Y_test, ax=ax)

   plt.plot([0,1], [0,1], ':',linewidth=1.4, c='k')

   plt.legend(bbox_to_anchor = (1.05, 0.6))


# In[8]:

#Precision-Recall curve

classifiers1 = [LR, NB, rf]

ax = plt.gca()

for i in classifiers1 :

   plot_precision_recall_curve(i, X_test, Y_test, ax=ax)

   plt.legend(bbox_to_anchor = (1.8, 0.6))

   for z in classifiers1 :
```

```
    precision, recall, thresholds = precision_recall_curve(Y_test, z.predict_proba(X_test)[:,1])
    area = auc(recall, precision)
    print("AUPRC",area)


# In[9]:
#Tune the model: Grid Search
from sklearn.model_selection import GridSearchCV
param_grid = {
    'bootstrap': [True],
    'max_depth': [80, 90, 100, 110],
    'max_features': [2, 3],
    'min_samples_leaf': [3, 4, 5],
    'min_samples_split': [8, 10, 12],
    'n_estimators': [100, 200, 300, 1000]
}
rf = RandomForestClassifier()
grid_search = GridSearchCV(estimator = rf, param_grid = param_grid, cv= 10, n_jobs = -1, verbose =
2, scoring='f1')
grid_search.fit(X_train, Y_train)
grid_search.best_estimator_


# In[9]:
#Performance
rf = RandomForestClassifier(bootstrap=True, ccp_alpha=0.0, class_weight=None,
                       criterion='gini', max_depth=80, max_features=3,
                       max_leaf_nodes=None, max_samples=None,
                       min_impurity_decrease=0.0, min_impurity_split=None,
                       min_samples_leaf=5, min_samples_split=12,
                       min_weight_fraction_leaf=0.0, n_estimators=1000,
                       n_jobs=None, oob_score=False, random_state=None,
                       verbose=0, warm_start=False).fit (X_train, Y_train)
```

```
#ROC Curve
classifiers = [rf]
ax = plt.gca()
for i in classifiers:
    plot_roc_curve(i, X_test, Y_test, ax=ax)
    plt.plot([0,1], [0,1], ':',linewidth=1.4, c='k')
    plt.legend(bbox_to_anchor = (1.05, 0.6))

# In[10]:
#Precision-Recall curve
classifiers1 = [rf]
ax = plt.gca()
for i in classifiers1 :
    plot_precision_recall_curve(i, X_test, Y_test, ax=ax)
    plt.legend(bbox_to_anchor = (1.8, 0.6))
# Compute Precision-Recall and plot curve
precision, recall, thresholds = precision_recall_curve(Y_test, rf.predict_proba(X_test)[:,1])
area = auc(recall, precision)
print("AUPRC",area)

# In[11]:
#Optimization of the Model
from sklearn.metrics import classification_report, confusion_matrix, ConfusionMatrixDisplay
from sklearn.metrics import recall_score
from sklearn.metrics import precision_score
from sklearn.metrics import f1_score
from sklearn.metrics import classification_report, confusion_matrix, ConfusionMatrixDisplay
#Metrics
y_pred_rf=rf.predict(X_test)
confusion = confusion_matrix(Y_test,y_pred_rf)
disp = ConfusionMatrixDisplay(confusion_matrix=confusion, display_labels=rf.classes_).plot()
```

```
plt.tick_params(axis=u'both', which=u'both',length=0)

precision= precision_score(Y_test, y_pred_rf)

recall=recall_score(Y_test, y_pred_rf)

F1= f1_score(Y_test, y_pred_rf)

print('F1-score:',F1)

print('Precision:',precision)

print('Recall:',recall)

total1=sum(sum(confusion))

accuracy1=(confusion[0,0]+confusion[1,1])/total1

print ('Accuracy : ', accuracy1)

specificity1 = confusion[0,0]/(confusion[0,0]+confusion[0,1])

print('Specificity : ', specificity1 )

sensitivity1 = confusion[1,1]/(confusion[1,0]+confusion[1,1])

print('Sensitivity : ', sensitivity1)


# In[12]:

#Tune the model: Best threshold

from numpy import arange

from numpy import argmax

from matplotlib import pyplot

from numpy import sqrt

# predict probabilities

yhat = rf.predict_proba(X_test)

# keep probabilities for the positive outcome only

yhat = yhat[:, 1]

# calculate roc curves

precision, recall, thresholds = precision_recall_curve(Y_test, yhat)

# convert to f score

fscore = (2 * precision * recall) / (precision + recall)

# locate the index of the largest f score

ix = argmax(fscore)
```

```
print('Best Threshold=%f, F-Score=%.3f' % (thresholds[ix], fscore[ix]))
# plot the roc curve for the model
no_skill = len(Y_test[Y_test==1]) / len(Y_test)
pyplot.plot([0,1], [no_skill,no_skill], linestyle='--', label='No Skill')
pyplot.plot(recall, precision, marker='.', label='Logistic')
pyplot.scatter(recall[ix], precision[ix], marker='o', color='black', label='Best')
# axis labels
pyplot.xlabel('Recall')
pyplot.ylabel('Precision')
pyplot.legend()
# show the plot
pyplot.show()


# In[15]:
#Set the new threshold
from sklearn.metrics import recall_score
from sklearn.metrics import precision_score
from sklearn.metrics import f1_score
from sklearn.metrics import balanced_accuracy_score
y_pred_new_threshold = (rf.predict_proba(X_test)[:,1]>=0.437613).astype(int)
#Metrics
confusion = confusion_matrix(Y_test,y_pred_new_threshold )
disp = ConfusionMatrixDisplay(confusion_matrix=confusion, display_labels=rf.classes_).plot()
plt.tick_params(axis=u'both', which=u'both',length=0)
precision= precision_score(Y_test, y_pred_new_threshold)
recall=recall_score(Y_test, y_pred_new_threshold)
F1= f1_score(Y_test, y_pred_new_threshold)
print('F1-score:',F1)
print('Precision/PPV:',precision)
print('Recall:',recall)
```

```
total1=sum(sum(confusion))
accuracy1=(confusion[0,0]+confusion[1,1])/total1
print ('Accuracy : ', accuracy1)
specificity1 = confusion[0,0]/(confusion[0,0]+confusion[0,1])
print('Specificity : ', specificity1 )
sensitivity1 = confusion[1,1]/(confusion[1,0]+confusion[1,1])
print('Sensitivity : ', sensitivity1)
NPV = confusion[0,0]/(confusion[0,0]+confusion[1,0])
print('NPV: ', NPV)


# In[7]:
#Features of importances
from matplotlib import pyplot
from matplotlib.pyplot import figure
from sklearn.ensemble import RandomForestClassifier
from sklearn.feature_selection import SelectFromModel
from sklearn.model_selection import cross_val_score
from sklearn.model_selection import train_test_split
jejuni = pd.read_csv ('Peak_matching_table_Ccoli_Cjejuni_Penny_Character_Mixed_IC_Cip.csv')
Y = jejuni['12']
X = jejuni.drop(columns=['12'])
clf1 = RandomForestClassifier(random_state=0)
clf1 = clf1.fit(X, Y)
model = SelectFromModel(clf, prefit=True)
features = X.columns.values
print(features[model.get_support()])
importances = clf1.feature_importances_
indices = np.argsort(importances)
# summarize feature importance
for i,v in enumerate(importances):
```

```
    print('Feature: %0d, Score: %.5f' % (i,v))
# plot feature importance
pyplot.bar([x for x in range(len(importances))], importances)
figure(figsize=(200, 10), dpi=600)
pyplot.show()
# Plot the feature importances of the forest
plt.figure(figsize=(10,20))
plt.title("Feature importances")
plt.barh(range(X.shape[1]), importances[indices],color="b",align="center")
plt.yticks(range(X.shape[1]), indices)
plt.ylim([-1, X.shape[1]])
plt.show()
```
